# Diagnostic value of MRI signs in differentiating Ewing sarcoma from osteomyelitis

**DOI:** 10.1177/0284185118774953

**Published:** 2018-05-09

**Authors:** Ömer Kasalak, Jelle Overbosch, Hugo JA Adams, Amelie Dammann, Rudi AJO Dierckx, Paul C Jutte, Thomas C Kwee

**Affiliations:** 1 Department of Radiology, Nuclear Medicine and Molecular Imaging, University Medical Center Groningen, University of Groningen, Groningen, The Netherlands; 2 Department of Radiology and Nuclear Imaging, Deventer Hospital, Deventer, The Netherlands; 3 Department of Orthopedics, University Medical Center Groningen, University of Groningen, Groningen, The Netherlands

**Keywords:** Ewing, magnetic resonance imaging, MRI, osteomyelitis

## Abstract

**Background:**

The value of magnetic resonance imaging (MRI) signs in differentiating Ewing sarcoma from osteomyelitis has not be thoroughly investigated.

**Purpose:**

To investigate the value of various MRI signs in differentiating Ewing sarcoma from osteomyelitis.

**Material and Methods:**

Forty-one patients who underwent MRI because of a bone lesion of unknown nature with a differential diagnosis that included both Ewing sarcoma and osteomyelitis were included. Two observers assessed several MRI signs, including the transition zone of the bone lesion, the presence of a soft-tissue mass, intramedullary and extramedullary fat globules, and the penumbra sign.

**Results:**

Diagnostic accuracies for discriminating Ewing sarcoma from osteomyelitis were 82.4% and 79.4% for the presence of a soft-tissue mass, and 64.7% and 58.8% for a sharp transition zone of the bone lesion, for readers 1 and 2 respectively. Inter-observer agreement with regard to the presence of a soft-tissue mass and the transition zone of the bone lesion were moderate (κ = 0.470) and fair (κ = 0.307), respectively. Areas under the receiver operating characteristic curve of the diameter of the soft-tissue mass (if present) were 0.829 and 0.833, for readers 1 and 2 respectively. Mean inter-observer difference in soft-tissue mass diameter measurement ± limits of agreement was 35.0 ± 75.0 mm. Diagnostic accuracies of all other MRI signs were all < 50%.

**Conclusion:**

Presence and size of a soft-tissue mass, and sharpness of the transition zone, are useful MRI signs to differentiate Ewing sarcoma from osteomyelitis, but inter-observer agreement is relatively low. Other MRI signs are of no value in this setting.

## Introduction

Ewing sarcoma is a highly aggressive bone and soft-tissue tumor (primarily bone) with a peak incidence in children and young adults (<30 years), while it is particularly rare among Asian and black populations (1,2). The annual incidence of Ewing sarcoma in the Western world has been reported to be around 2.93/1,000,000 cases (3). Ewing sarcoma in bone may mimic osteomyelitis clinically (both may present with fever, increased serum inflammatory markers, and bone pain) and on imaging examinations (both may present with aggressive periosteal reaction, cortical destruction, and articular involvement) (4). In fact, it has been reported that up to 50% of subacute osteomyelitis cases in children are confused with tumor (5). Timely and accurate differentiation between these two entities, however, is important because treatment and outcome are completely different (1,6).

Only two previous studies have specifically focused on the important differentiation between Ewing sarcoma and osteomyelitis (7,8). In a study by Henninger et al. (7) that investigated six magnetic resonance imaging (MRI) features in 18 patients with osteomyelitis and ten patients with Ewing sarcoma, a sharp and defined margin of the bone lesion was reported to be the most significant feature that differentiated Ewing sarcoma from osteomyelitis. However, in another study by McCarville et al. (8) that evaluated 36 radiographic and MRI parameters in 32 patients with osteomyelitis and 31 patients with Ewing sarcoma, it was reported that most individuals with Ewing sarcoma or osteomyelitis had a wide transition zone on MRI and that this feature did not predict the diagnosis (8). The latter study also reported that a soft-tissue mass was more likely to occur in association with Ewing sarcoma than with osteomyelitis and that no other clinical or imaging features (except ethnicity) were predictive of prognosis (8). Besides the partially conflicting results between Henninger et al.’s and McCarville et al.’s studies (7,8), one of the main limitations of these two studies is that they only included Ewing sarcoma and osteomyelitis cases, and that other differential diagnostic entities were excluded (7,8). This limits the clinical applicability of their results as this is often not the situation which clinicians face. Therefore, further research is necessary to clarify the true diagnostic value of the MRI signs that were proposed by Henninger et al. and McCarville et al. (7,8). Expanding on these previous studies, it can be hypothesized that the size of the soft-tissue mass adjacent to the involved bone (if present) may also be of importance, in that the soft-tissue mass associated with Ewing sarcoma may show more expansion eccentric to the long axis of the bone than in osteomyelitis. Other potentially useful MRI signs in this setting are the so-called “penumbra sign” (i.e. a peripheral layer surrounding a cavity in either the bone marrow or adjacent soft tissues that is hyperintense on unenhanced T1-weighted (T1W) images and enhances intensely after administration of gadolinium (9)), and the presence of intramedullary or extramedullary fat globules (i.e. one or more localized fat collections in the bone marrow or adjacent soft-tissue (10)), all of which have been suggested to indicate osteomyelitis. However, the penumbra sign was not seen in any of the osteomyelitis cases in Henninger et al.’s study (7) and in only two osteomyelitis cases in McCarville et al.’s study (8). Furthermore, the diagnostic value of the presence of intra- or extramedullary fat globules in differentiating Ewing sarcoma from osteomyelitis has never been investigated.

The purpose of this study was therefore to investigate the diagnostic value of several potentially useful MRI signs in differentiating Ewing sarcoma from osteomyelitis in patients who presented with a bone lesion of unknown nature with a differential diagnosis that included both Ewing sarcoma or osteomyelitis.

## Material and Methods

### Study design and patients

This retrospective study was approved by the local institutional review board, and the requirement for written informed consent was waived. The Picture Archiving and Communication System (PACS) of a tertiary referral center for bone tumors was searched for all patients who underwent MRI between 2010 and 2016 because of a bone lesion of unknown nature with a differential diagnosis that included both Ewing sarcoma and osteomyelitis. Inclusion criteria for this study were: patients aged 30 years and younger; presence of a bone lesion on radiography of unknown nature with both Ewing sarcoma and osteomyelitis in the differential diagnosis; availability of an MRI examination of the bone lesion in the PACS system; and biopsy (either percutaneous computed tomography [CT]- or ultrasound-guided or open surgical); or at least six months follow-up as reference standard. Patients who were eventually diagnosed with a disease other than Ewing sarcoma or osteomyelitis were also eligible for inclusion. Exclusion criteria for this study were: biopsy or surgery before MRI was performed; pathologically or microbiologically proven diagnosis before MRI was performed; extra-osseous Ewing sarcoma; craniospinal Ewing sarcoma; presence of metastatic disease on other imaging modalities before MRI was performed; and patients with clinical findings highly suggestive of osteomyelitis before MRI was performed (such as bacteremia, history of osteomyelitis, cutaneous defects/fistulae, recent musculoskeletal surgery, and presence of osteosynthetic or prosthetic material).

### MRI acquisition

MRI scans were performed using clinical 1.5-T MRI systems. Eleven of 41 patients who were eventually included had undergone MRI before referral to our hospital. Therefore, MRI protocols were not uniform. Nevertheless, all included patients were scanned with unenhanced T1-weighted (T1W), T2-weighted (T2W), and fat-suppressed T2W sequences. Gadolinium-enhanced sequences were acquired in 36 of 41 patients (five patients who were eventually diagnosed with osteomyelitis did not undergo gadolinium-enhanced MRI). Applied slice thicknesses were in the range of 0.9–4.0 mm. Sequences or reconstructed images (in case a three-dimensional isotropic MRI sequence was acquired) were oriented in at least two directions with regard to the involved bone.

### MRI evaluation

MRI datasets were reviewed in a random order by two radiologists (ÖK and JO, with four years and nine years of experience in musculoskeletal MRI, respectively) using a PACS workstation (Carestream Vue PACS version 11.4.1.1102, Carestream Health, Inc, Rochester, NY, USA). Both readers knew the age and gender of each patient and were aware of the fact that MRI was performed because of a bone lesion of unknown nature and a differential diagnosis of both Ewing sarcoma and osteomyelitis. However, both readers were blinded to each other’s assessments, original MRI reports, final diagnosis, and other clinical, pathological, and follow-up findings. Radiographs, CT scans, and other imaging tests performed before MRI, if available, were not reviewed during MRI evaluation. MRI scans were evaluated with regard to the transition zone of the bone lesion (sharp vs. unsharp on T1W images in comparison to fat-suppressed T2W images) (7), presence of a soft-tissue mass (i.e. a lobular or infiltrative structure located in the adjacent soft-tissue that contains solid material [is not completely fluid on a water-sensitive sequence] and has areas that enhance after gadolinium administration) (8), the maximum diameter of the soft-tissue mass perpendicular to the long axis of the involved bone (caliper measurement), the presence of the penumbra sign (i.e. a peripheral layer surrounding a cavity in either the bone marrow or adjacent soft tissues that is hyperintense on unenhanced T1W images and enhances intensely after administration of gadolinium) (9), and the presence of intramedullary and/or extramedullary fat globules (defined as T1-hyperintense foci within the involved bone marrow or adjacently involved soft tissue) (10). In case of two lesions in the MRI field of view in the same patient, only the largest lesion was analyzed. The two readers were provided with examples of MRI scans that were published in previous studies to facilitate the interpretation of these MRI signs (7–10).

### Reference standard

Percutaneous CT- or ultrasound-guided biopsy with needle sizes in the range of 9–18 gauge (depending on the preference of the attending radiologist as determined in each individual patient) or open surgical biopsy (performed by an orthopedic surgeon) followed by histopathological examination (performed by a pathologist with expertise in bone tumors) and tissue cultures (interpreted by a microbiologist) served as reference standard. If biopsy was not performed or inconclusive, clinical and imaging follow-up of at least six months served as reference standard.

### Statistical analysis

Diagnostic accuracy (as global measure of diagnostic performance), sensitivity, specificity, positive predictive value (PPV), and negative predictive value (NPV) of each of the investigated MRI signs for the discrimination between Ewing sarcoma from osteomyelitis were calculated (excluding other diagnostic entities), along with 95% confidence intervals (CIs). Receiver operating characteristic (ROC) analysis was performed to determine the value of the diameter of the soft-tissue mass in patients who presented with a soft-tissue mass adjacent to the involved bone. The area under the ROC curve (AUC) and optimal cut-off value with corresponding sensitivity and specificity were calculated. The same analyses were then repeated for Ewing sarcoma or other malignancy vs. osteomyelitis or other benign lesions. Inter-observer agreement with regard to the evaluated MRI signs was analyzed using the unweighted κ statistic, defined as poor (<0.2), fair (>0.2 to ≤0.4), moderate (>0.4 to ≤0.6), good (>0.6 to ≤ 0.8), and very good (>0.8 to ≤1) agreement. Inter-observer agreement with regard to the measurement of the soft-tissue mass diameter was determined as mean absolute difference (bias) and 95% CI of the mean difference (limits of agreement) according to the methods of Bland and Altman (11). Statistical analyses were executed using MedCalc version 17.2 Software (MedCalc, Mariakerke, Belgium).

## Results

### Patients

A total of 84 patients were potentially eligible for inclusion. However, 25 patients were excluded because of either already proven or clinical findings highly suggestive of osteomyelitis, five patients were excluded because of extra-osseous Ewing sarcoma, five patients were excluded because of lack of an MRI scan in the PACS, three patients were excluded because of craniospinal Ewing sarcoma, three patients with Ewing sarcoma were excluded because of presence of metastatic disease on other imaging modalities before MRI was performed, and two patients were excluded because of pathologically proven diagnosis before MRI was performed (one Ewing sarcoma and one eosinophilic granuloma). Thus, 41 patients (19 boys/men, 22 girls/women, all of Caucasian ethnicity; mean age = 11.2 ± 5.3 years; age range = 0–25 years) were finally included. The bone lesions were located in the femur (n = 9), tibia (n = 9), pelvic bone (n = 8), clavicle (n = 5), humerus (n = 2), radius (n = 2), fibula (n = 2), clavicle and rib (n = 1), rib (n = 1), femur and tibia (n = 1), and talus (n = 1). The basic characteristics of included patients are shown in Table 1.

**Table 1.** Basic characteristics of included patients.

Characteristic	Ewing sarcoma	Osteomyelitis*	Other malignancy^†^	Other benign lesions^‡^
Patients (n)	14	20	3	4
Mean age ± SD (years)	13.4 ± 4.9	10.6 ± 5.8	7.7 ± 3.2	8.8 ± 2.5
Age range (years)	5–25	0–23	4–10	5–12
Male/female (n)	8/6	9/11	0/3	2/2
Affected bones (n)				
Femur	2	5	1	1
Tibia	3	4	–	2
Pelvis	5	2	1	–
Clavicle	1	4	–	–
Humerus	1	–	1	–
Radius	–	1	–	1
Fibula	1	1	–	–
Clavicle and rib	–	1	–	–
Rib	1	–	–	–
Femur and tibia	–	1	–	–
Talus	–	1	–	–
Locations within long bones^§^				
Diaphysis only	2	5		2
Metaphysis only		3	–	
Diaphysis and metaphysis	3	2	–	2
Metaphysis and epiphysis	–	2	–	–
Dia-, meta-, and epiphysis	3	5	2	–
Involvement of epiphysial plate**	2	3	2	–

*Bacterial osteomyelitis (n = 10) and non-bacterial osteitis (n = 10).

^†^Osteosarcoma (n = 2) and acute lymphoblastic leukemia (n = 1).

^‡^Eosinophilic granuloma (n = 2), aneurysmatic bone cyst (n = 1), and stress fracture (n = 1).

^§^In patients with bone lesions in the clavicle, humerus, radius, femur, tibia, and fibula.

**In patients with an unfused epiphyseal plate of the involved bone.

### Reference standard

Diagnosis was based on percutaneous CT- or ultrasound-guided biopsy in 25 patients (Ewing sarcoma: n = 8; osteomyelitis: n = 10; other malignancy: n = 3; other benign lesion: n = 4), open surgical biopsy in 12 patients (Ewing sarcoma: n = 6; osteomyelitis: n = 6), and clinical and imaging follow-up (minimum follow-up time = 18 months) without biopsy in four patients (osteomyelitis: n = 4). Final diagnoses were Ewing sarcoma in 14 patients, osteomyelitis in 20 patients (of which ten bacterial osteomyelitis and ten non-bacterial osteitis), osteosarcoma in two patients, eosinophilic granuloma in two patients, acute lymphoblastic leukemia (without blastic cells in peripheral blood tests) in one patient, aneurysmatic bone cyst in one patient, and stress fracture in one patient.

### Diagnostic performance MRI signs

**Fig. 1.**
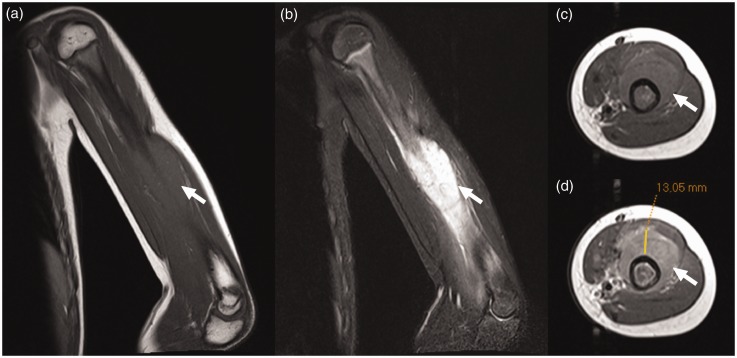
A 9-year-old girl with Ewing sarcoma in the left humerus. Coronal T1W (a), coronal fat-suppressed T2W (b), axial T1W (c), and axial gadolinium-enhanced (d) images with caliper measurement (D) show a lesion in the left humerus with a soft-tissue mass (arrows). Both observers reported the presence of a soft-tissue mass.

**Fig. 2.**
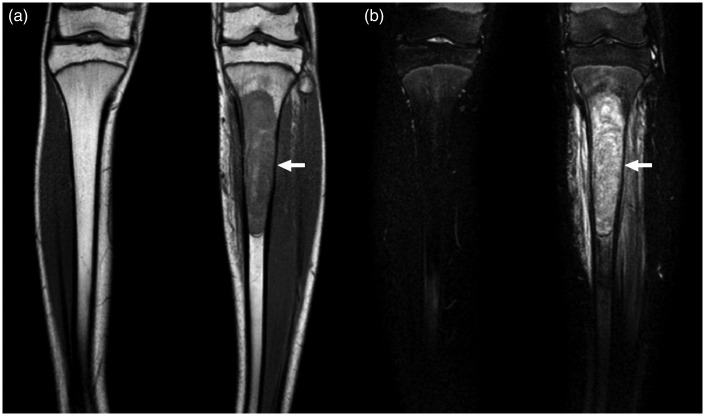
A 15-year-old boy with Ewing sarcoma of the left tibia. Coronal T1W (a) and coronal fat-suppressed T2W images (b) show the lesion in the proximal left tibia. Both observers reported the presence of a sharp transition zone of the bone lesion.

**Fig. 3.**
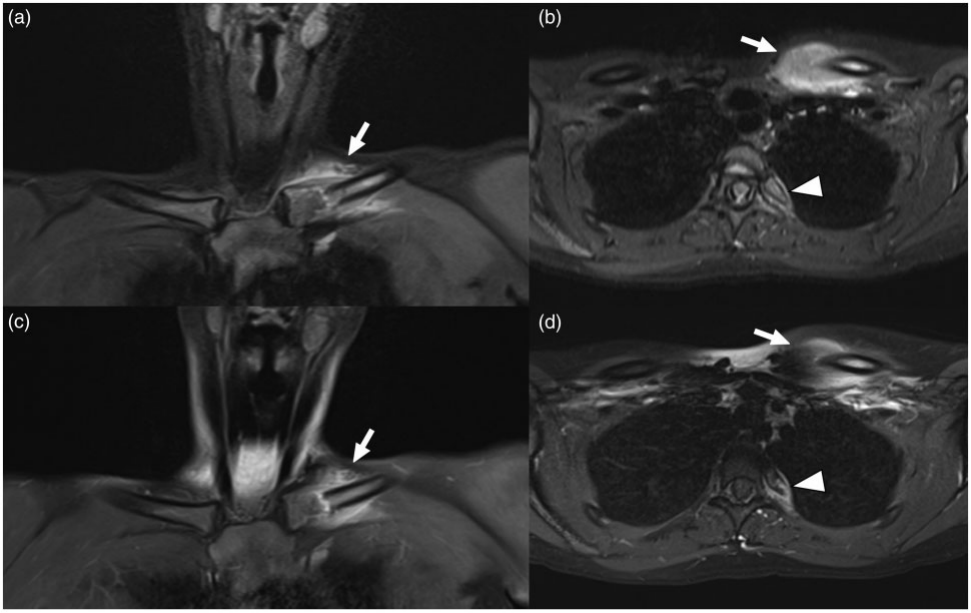
A 15-year-old girl with bacterial osteomyelitis (*Propionibacterium acnes*) of the left clavicle and left proximal fourth rib. Coronal (a) and axial (b) fat-suppressed T2W images and coronal (c) and axial (d) gadolinium-enhanced images shown the lesion in the medial left clavicle (arrows) and proximal left fourth rib (arrowheads). Both observers reported the presence of a soft-tissue mass.

**Fig. 4.**
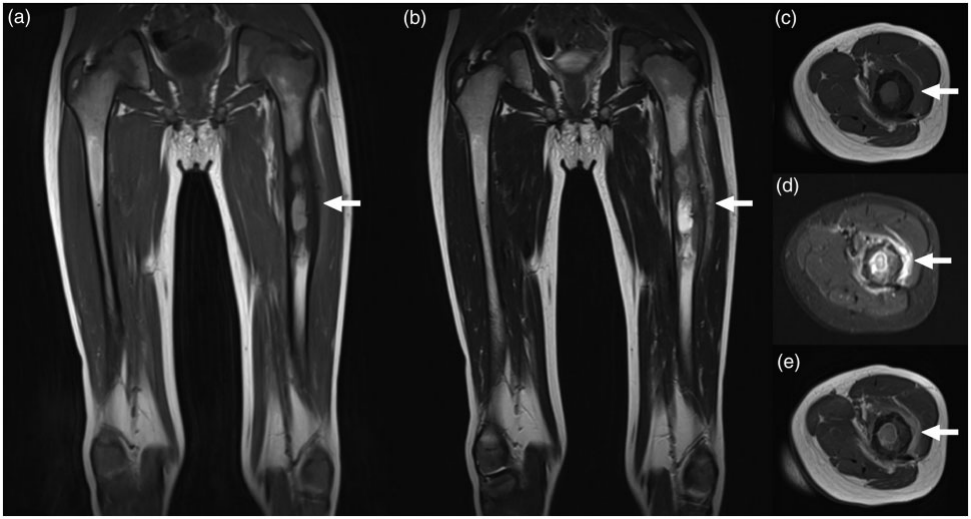
A five-year-old boy with osteomyelitis of the left femur (the causative agent remained unclear). Coronal T1W (a), coronal T2W (b), axial T1W (c), axial fat-suppressed T2W (d), and axial gadolinium-enhanced T1W (e) images show the lesion in the left femur (arrows). Observer 1 reported the transition zone of the bone lesion to be unsharp and the lack of a soft-tissue mass, whereas observer 2 reported the opposite for both items.

Measures of diagnostic performance are shown in Tables 2 and 3. Diagnostic accuracies for discriminating Ewing sarcoma from osteomyelitis were 64.7% and 58.8% for a sharp transition zone of the bone lesion, and 82.4% and 79.4% for the presence of a soft-tissue mass, for readers 1 and 2, respectively. Diagnostic accuracies for discriminating Ewing sarcoma or malignancy from osteomyelitis or other benign lesions were 68.3% and 58.5%, for a sharp transition zone of the bone lesion, and 80.5% and 75.6% for the presence of a soft-tissue mass, for readers 1 and 2, respectively. Representative examples are shown in Figs. 1–4. Diagnostic accuracies for intramedullary fat globules, extramedullary fat globules, and the penumbra sign were all <50%.

**Table 2.** Diagnostic performance of MRI signs in discriminating Ewing sarcoma from osteomyelitis (excluding all other diagnostic entities).

MRI sign	Reader	Diagnostic accuracy* (%)	Sensitivity* (%)	Specificity* (%)	PPV* (%)	NPV* (%)
Sharp transition zone bone lesion	1	64.7 (47.9–78.5)	21.4 (7.6–47.6)	95.0 (76.4–99.1)	75.0 (30.1–95.4)	63.3 (45.5–78.1)
	2	58.8 (42.2–73.6)	42.9 (21.4–67.4)	70.0 (48.1–85.5)	50.0 (25.4–74.6)	63.6 (43.0–80.3)
Presence of soft-tissue mass	1	82.4 (66.5–91.7)	85.7 (60.1–96.0)	80.0 (58.4–91.9)	75.0 (50.5–89.8)	88.9 (67.2–96.9)
	2	79.4 (63.2–89.7)	92.9 (68.5–98.7)	70.0 (48.1–85.5)	68.4 (46.0–84.6)	93.3 (70.2–98.8)
Absence of intramedullary fat globules	1	41.2 (26.4–57.8)	100 (78.5–100)	0 (0–16.1)	41.2 (26.4–57.8)	NA^†^
	2	32.4 (19.1–49.2)	78.6 (52.4–92.4)	0.0 (0.0–16.1)	35.5 (21.1–53.1)	0.0 (0.0–56.2)
Absence of extramedullary fat globules	1	41.2 (26.4–57.8)	100 (78.5–100)	0 (0–16.1)	41.2 (26.4–57.8)	NA^†^
	2	41.2 (26.4–57.8)	100 (78.5–100)	0 (0–16.1)	41.2 (26.4–57.8)	NA^†^
Absence of penumbra sign	1	41.2 (26.4–57.8)	100 (78.5–100)	0 (0–16.1)	41.2 (26.4–57.8)	NA^†^
	2	32.4 (19.1–49.2)	78.6 (52.4–92.4)	0.0 (0.0–16.1)	35.5 (21.1–53.1)	0.0 (0.0–56.2)

*95% CIs between parentheses.

^†^Not available; insufficient number of categories to perform test.

**Table 3.** Diagnostic performance of MRI signs in discriminating Ewing sarcoma or malignancy from osteomyelitis or other benign lesions.

MRI sign	Reader	Diagnostic accuracy* (%)	Sensitivity* (%)	Specificity* (%)	PPV* (%)	NPV* (%)
Sharp transition zone bone lesion	1	68.3 (53.0–80.4)	29.4 (13.3–53.1)	95.8 (79.8–99.3)	83.3 (43.7–97.0)	65.7 (49.2–79.2)
	2	58.5 (43.4–72.2)	47.1 (26.2–69.0)	66.7 (46.7–82.0)	50.0 (28.0–72.0)	64.0 (44.5–79.8)
Presence of soft-tissue mass	1	80.5 (66.0–89.8)	82.4 (59.0–93.8)	79.2 (59.5–90.8)	73.7 (51.2–88.2)	86.4 (66.7–95.3)
	2	75.6 (60.7–86.2)	88.2 (65.7–96.7)	66.7 (46.7–82.0)	65.2 (44.9–81.2)	88.9 (67.2–96.9)
Absence of intramedullary fat globules	1	41.5 (27.8–56.6)	100 (81.6–100)	0 (0–13.8)	41.5 (27.8–56.6)	NA^†^
	2	31.7 (19.6–47.0)	76.5 (52.7–90.4)	0 (0.0–13.8)	35.1 (21.8–51.2)	0.0 (0.0–49.0)
Absence of extramedullary fat globules	1	41.5 (27.8–56.6)	100 (81.6–100)	0 (0–13.8)	41.5 (27.8–56.6)	NA^†^
	2	41.5 (27.8–56.6)	100 (81.6–100)	0 (0–13.8)	41.5 (27.8–56.6)	NA^†^
Absence of penumbra sign	1	43.9 (29.9–59.0)	100 (81.6–100)	4.2 (1.0–20.2)	42.5 (28.5–57.8)	100 (20.7–100)
	2	34.2 (21.6–49.5)	82.4 (59.0–93.8)	0.0 (0.0–13.8)	36.8 (23.4–52.7)	0.0 (0.0–56.2)

*95% CIs in parentheses.

^†^Not available; insufficient number of categories to perform test.

### Diagnostic performance diameter soft-tissue mass

Reader 1 judged a soft-tissue mass to be present in 19 patients, of whom 12 with Ewing sarcoma (mean diameter = 31 ± 23 mm; range = 9–79 mm), four with osteomyelitis (mean diameter ± SD = 15 ± 20 mm; range = 4–44 mm), two with another malignancy (diameters = 13 and 34 mm for two osteosarcomas), and one with another benign lesion (diameter = 6 mm for one eosinophilic granuloma). AUC of the diameter of the soft-tissue mass in discriminating Ewing sarcoma from osteomyelitis was 0.792 (95% CI = 0.520–0.948) and an optimal cut-off diameter of 6 mm yielded sensitivity and specificity values of 100% (95% CI = 73.4–100) and 75.0% (95% CI = 20.3–95.9). AUC of the diameter of the soft-tissue mass in discriminating Ewing sarcoma or malignancy from osteomyelitis or other benign lesions was 0.829 (95% CI = 0.588–0.958) and an optimal cut-off diameter of 6 mm yielded sensitivity and specificity values of 100% (95% CI = 76.7–100) and 80.0% (95% CI = 28.8–96.7).

Reader 2 judged a soft-tissue mass to be present in 23 patients, of whom 13 with Ewing sarcoma (mean diameter ± SD = 65 ± 41 mm; range = 7–175 mm), six with osteomyelitis (mean diameter ± SD = 33 ± 32 mm; range = 9–95 mm), two with another malignancy (diameters = 54 and 75 mm for two osteosarcomas), and two with another benign lesion (diameters = 28 and 29 mm for two eosinophilic granulomas). AUC of the diameter of the soft-tissue mass in discriminating Ewing sarcoma from osteomyelitis (excluding all other diagnostic entities) was 0.795 (95% CI = 0.550–0.941) and an optimal cut-off diameter of 36 mm yielded sensitivity and specificity values of 84.6% (95% CI = 54.5–97.6) and 83.3% (95% CI = 36.1–97.2). AUC of the diameter of the soft-tissue mass in discriminating Ewing sarcoma or malignancy from osteomyelitis or other benign lesions was 0.833 (95% CI = 0.620–0.953) and an optimal cut-off diameter of 36 mm yielded sensitivity and specificity values of 86.7% (95% CI = 59.5–98.0) and 87.5% (95% CI = 47.4–97.9).

### Inter-observer agreement

Inter-observer agreement results of MRI signs are shown in Table 4. Inter-observer agreements with regard to the transition zone of the bone lesion and the presence of a soft-tissue mass were fair and moderate with κ values of 0.307 and 0.470, respectively (Figs. 1–4). However, inter-observer agreement with regard to the assessment of intramedullary fat globules and the penumbra sign was poor, with κ values of 0.000 and −0.038, respectively. Inter-observer agreement with regard to the assessment of extramedullary fat globules could not be assessed due to the lack of positive scores. In 15 patients, both observers 1 and 2 judged a soft-tissue mass to be present. In these 15 patients (who all had undergone gadolinium-enhanced MRI), mean inter-observer difference in soft-tissue mass diameter measurement ± limits of agreement was 35.0 ± 75.0 mm.

**Table 4.** Inter-observer agreement results of MRI signs.

MRI sign	Kappa*
Sharp transition zone bone lesion	0.307 (−0.0227–0.637)
Presence of soft-tissue mass	0.470 (0.202–0.738)
Absence of intramedullary fat globules	0.000 (−0.0931–0.931)
Absence of extramedullary fat globules	NA^†^
Absence of penumbra sign	–0.038 (−1.004–0.928)

*95% CIs between parentheses.

^†^Not available; insufficient number of categories to perform test.

## Discussion

The results of this study show that the presence of a soft-tissue mass is the most valuable MRI sign for discriminating Ewing sarcoma from osteomyelitis, with diagnostic accuracies of around 80%. This finding is also applicable to the discrimination of Ewing sarcoma or other malignancy from osteomyelitis or other benign lesions. The transition zone of the bone lesion was only moderately useful with diagnostic accuracies of around 60%. Diagnostic accuracies for intramedullary and extramedullary fat globules and the penumbra sign were all < 50%. None of the evaluated MRI signs reached a consistently high PPV or NPV to reliably rule in or rule out Ewing sarcoma. Yet another important finding is that in patients with a soft-tissue mass adjacent to the involved bone, additional soft-tissue mass diameter measurements perpendicular to the long axis of the involved bone were found to be useful in differentiating Ewing sarcoma from osteomyelitis, with larger diameters found in Ewing sarcoma. Note that osteomyelitis not uncommonly presents with an adjacent soft-tissue mass (in around 25% of cases in the present study) (7,8) and that the size of the soft-tissue mass may thus be diagnostically helpful. However, no cut-off values can currently be recommended, because inter-observer agreement was low with mean difference in soft-tissue mass diameter measurement ± limits of agreement of 35.0 ± 75.0 mm. Inter-observer agreement with regard to the presence of a soft-tissue mass and transition zone was also relatively low with fair to moderate κ values.

Both the present study and two previous studies on this topic (7,8) included patients with a bone lesion of unknown nature with a differential diagnosis of both Ewing sarcoma and osteomyelitis, but only the former did not exclude patients who were eventually diagnosed with another entity. Moreover, the present study used strict enrollment criteria to only include patients in whom the diagnosis was still unclear while excluding patients in whom the diagnosis was already proven or suspected based on pathological, clinical, or other imaging findings. Therefore, the present results may have greater clinical applicability. Nevertheless, the studies by Henninger et al. (7) and McCarville et al. (8) also reported relatively high diagnostic accuracies for the presence of a soft-tissue mass in diagnosing Ewing sarcoma of 78.6% (95% CI = 60.1–89.8) and 75.5% (95% CI = 62.4–85.1), respectively. The biggest discrepancy is that Henninger et al. (7) reported the transition zone of the bone lesion to have a diagnostic accuracy of 100% (95% CI = 87.9–100), while McCarville et al. (8) reported a diagnostic accuracy of only 45.3% (95% CI = 32.4–58.6) for this sign, the latter more in line with the findings of the present study. Yet another discrepancy between Henninger et al.’s study (7) and the present study is that the former reported that overall MRI inter-observer reliability was good (with a κ value of 0.7590), while the latter found a relatively low inter-observer agreement. The penumbra sign and the presence of intramedullary or extramedullary fat globules have also been proposed as specific signs for osteomyelitis in previous studies in which only osteomyelitis patients were included, and these signs have also been suggested as potentially useful for differentiating osteomyelitis from malignancy (9,10). However, none of these signs were found to be of diagnostic value in the present study.

This study had several limitations. First, the results of this study are only applicable to patients aged ≤30 years (this upper limit was chosen because the vast majority of Ewing sarcomas occur below this age (1,2)) who present with a bone lesion of unknown nature with both Ewing sarcoma and osteomyelitis in the differential diagnosis. The findings are not applicable to patients aged >30 years or with a bone lesion in which Ewing and osteomyelitis are not in the differential diagnosis based on clinical or radiographic findings. Second, radiographs were not analyzed, but previous work has already shown that radiography has no independent diagnostic value compared to MRI in this setting (8). Third, MRI protocols were not uniform, since 11 of 41 patients had undergone MRI elsewhere before referral to our hospital; this is a weakness in the present study but reflects clinical practice. Fourth, the relatively low inter-observer agreement and particularly the optimal cut-off of the size of the soft-tissue mass for discriminating Ewing sarcoma from osteomyelitis, require further optimization and investigation. Fifth, MRI scans were interpreted by two musculoskeletal radiologists who work in a tertiary referral center for bone tumors. Further research should also investigate the diagnostic performance and inter-observer agreement of MRI among general radiologists who less frequently encounter bone tumors in this patient population.

In conclusion, the presence and size of a soft-tissue mass, and to a lesser extent the sharpness of the transition zone, are useful MRI signs to differentiate Ewing sarcoma from osteomyelitis, but inter-observer agreement is relatively low. Other MRI signs are of no value in this setting.
